# Association Between Crowdsourced Health Care Facility Ratings and Mortality in US Counties

**DOI:** 10.1001/jamanetworkopen.2021.27799

**Published:** 2021-10-19

**Authors:** Daniel C. Stokes, Arthur P. Pelullo, Nandita Mitra, Zachary F. Meisel, Eugenia C. South, David A. Asch, Raina M. Merchant

**Affiliations:** 1 Department of Medicine, David Geffen School of Medicine, University of California, Los Angeles; 2 Center for Digital Health, Penn Medicine, University of Pennsylvania, Philadelphia; 3 Department of Biostatistics, Epidemiology, and Informatics, University of Pennsylvania, Philadelphia; 4 Leonard Davis Institute of Health Economics, University of Pennsylvania, Philadelphia; 5 Department of Emergency Medicine, Perelman School of Medicine, University of Pennsylvania, Philadelphia; 6 Urban Health Lab, Perelman School of Medicine, University of Pennsylvania, Philadelphia; 7 Division of General Internal Medicine, Perelman School of Medicine, University of Pennsylvania, Philadelphia

## Abstract

**Importance:**

Mortality across US counties varies considerably, from 252 to 1847 deaths per 100 000 people in 2018. Although patient satisfaction with health care is associated with patient- and facility-level health outcomes, the association between health care satisfaction and community-level health outcomes is not known.

**Objective:**

To examine the association between online ratings of health care facilities and mortality across US counties and to identify language specific to 1-star (lowest rating) and 5-star (highest rating) reviews in counties with high vs low mortality.

**Design, Setting, and Participants:**

This retrospective population-based cross-sectional study examined reviews and ratings of 95 120 essential health care facilities across 1301 US counties. Counties that had at least 1 essential health care facility with reviews available on Yelp, an online review platform, were included. Essential health care was defined according to the 10 essential health benefits covered by Affordable Care Act insurance plans.

**Main Outcomes and Measures:**

The mean rating of essential health care facilities was calculated by county from January 1, 2015, to December 31, 2019. Ratings were on a scale of 1 to 5 stars, with 1 being the worst rating and 5 the best. County-level composite measures of health behaviors, clinical care, social and economic factors, and physical environment were obtained from the University of Wisconsin School of Medicine and Public Health County Health Rankings database. The 2018 age-adjusted mortality by county was obtained from the Centers for Disease Control and Prevention Wide-ranging Online Data for Epidemiological Research database. Multiple linear regression analysis was used to estimate the association between mean facility rating and mortality, adjusting for county health ranking variables. Words with frequencies of use that were significantly different across 1-star and 5-star reviews in counties with high vs low mortality were identified.

**Results:**

The 95 120 facilities meeting inclusion criteria were distributed across 1301 of 3142 US counties (41.4%). At the county level, a 1-point increase in mean rating was associated with a mean (SE) age-adjusted decrease of 18.05 (3.68) deaths per 100 000 people (*P* < .001). Words specific to 1-star reviews in high-mortality counties included *told*, *rude*, and *wait*, and words specific to 5-star reviews in low-mortality counties included *Dr*, *pain*, and *professional*.

**Conclusions and Relevance:**

This study found that, at the county level, higher online ratings of essential health care facilities were associated with lower mortality. Equivalent online ratings did not necessarily reflect equivalent experiences of care across counties with different mortality levels, as evidenced by variations in the frequency of use of key words in reviews. These findings suggest that online ratings and reviews may provide insight into unequal experiences of essential health care.

## Key Points

**Question:**

Are online ratings of essential health care facilities associated with mortality at the county level?

**Findings:**

In this cross-sectional study of reviews and ratings of 95 120 essential health care facilities across 1301 US counties, counties with facilities with a 1-point higher mean rating on an online review platform had 18.1 fewer age-adjusted deaths per 100 000 people. Natural language processing analyses found significant differences in online review content between counties with high vs low mortality.

**Meaning:**

This study’s findings suggest that online reviews of essential health care facilities may reveal local health inequities and provide insights to improve local health care satisfaction.

## Teaser

This cross-sectional study examines the association between online ratings of health care facilities and mortality across US counties and identifies words specific to reviews with 1-star (lowest) and 5-star (highest) ratings in counties with low vs high mortality.

## Introduction

Mortality varies greatly by US county, ranging from 252 to 1847 deaths per 100 000 people in 2018.^1^ Differences in all-cause mortality by county have increased over time.^2^ When measured at the county level, certain health behaviors, aspects of clinical care, social and economic factors, and features of the physical environment are associated with county-level mortality.^3^ Traditional population-level measures of the quality of clinical care include items related to access (eg, number of primary care physicians) and outcomes (eg, preventable hospital stays).^3^ Patient experience and satisfaction with health care are increasingly recognized as important measures of health care quality, but data on these factors are less widely collected.^4^ Favorable evaluations of health care are associated with both patient-level outcomes, such as improved medication adherence, and facility-level outcomes, such as lower mortality.^5,6^ Patient experience might also be associated with county-level mortality, but those associations have not yet been well explored.

Crowdsourced online review sites can provide insight into patient experiences with local health care facilities and practitioners and cover a broader range of life-prolonging medical services compared with patient surveys conducted by the Centers for Medicare & Medicaid Services (CMS).^7,8,9,10,11,12,13^ The CMS survey provides patients with the opportunity to rate hospitals, home health care services, and dialysis facilities but excludes other services providing life-prolonging care.^14^ The 2010 Affordable Care Act (ACA) identified 10 essential health benefits extending beyond those services covered by CMS patient surveys and including, for instance, ambulatory services and mental health and substance use disorder services.^15^ Yelp, an online review platform, has been used to evaluate these and other services, with the added advantage of including both ratings and narrative descriptions of patients’ care experiences.^7,9,10,13^ Studies of patient reviews of health care facilities on this platform have reported consistency between CMS patient survey findings and qualitative and quantitative findings from crowdsourced reviews (when such comparisons have been possible).^7,8,16^ Crowdsourced online ratings have also been associated with facility-level health outcomes; facilities with a greater percentage of high ratings in the online reviews or CMS surveys had lower mortality associated with pneumonia and myocardial infarction and lower readmission rates for pneumonia, myocardial infarction, and heart failure.^17^ A recent analysis of skilled nursing facilities found that facilities with the highest ratings on both Yelp and the CMS Nursing Home Compare sites had 2.0% lower rehospitalization rates than those with the lowest ratings.^7^

To explore the hypothesis that patient ratings of essential health care facilities are associated with health outcomes at a community level, we examined the association between mean ratings for essential health care facilities and county-level mortality using the ACA definition of essential health benefits.^15^ To explore potential factors associated with patient satisfaction in counties with markedly different mortality, we identified common words most different in frequency among 5-star (highest) and 1-star (lowest) reviews of facilities in high-mortality vs low-mortality counties.

## Methods

This study was approved by the institutional review board of the University of Pennsylvania and deemed exempt from informed consent because of the use of publicly available data. The study followed the Strengthening the Reporting of Observational Studies in Epidemiology (STROBE) reporting guideline for cross-sectional studies.^18^

### Data and Sample

We identified all US counties with at least 1 essential health care facility with reviews available on the review platform, in 2018, the most recent year for which county-level mortality data were available. Health facilities on the platform are assigned to 1 or more of 159 categories, including categories likely to provide care that meets the ACA definition of essential health benefits (eg, nephrologists or emergency medicine) and categories unlikely to provide care that meets the ACA definition (eg, medical transportation or cryotherapy).^15^ Two authors (D.C.S. and R.M.M.) independently matched categories on the platform to ACA essential health benefits. In the few cases (<2%) of discrepancy in matching, the 2 authors were able to reach consensus on the best match through further discussion. Of 159 categories, 79 were matched to an ACA health benefit category (eTable 1 in the Supplement). Three ACA benefit categories—ambulatory patient services, hospitalization, and preventive or wellness services and chronic disease management—were grouped because many of the platform’s categories (eg, doctors, dermatologists, and internal medicine) could apply to any or all of these 3 benefits. Given that many facility and practitioner categories matched multiple ACA health benefits and that a single facility could be linked to multiple categories, we chose to analyze facilities across the 79 matched categories in aggregate.

Facilities were included if they could be categorized in 1 of the 79 matched platform’s categories and if they were open in 2018, with open defined as having at least 1 review in 2018 or at least 1 review both before and after 2018. For included facilities, review data were gathered for the 5-year span from January 1, 2015, to December 31, 2019. Previous analyses have found that ratings of health care facilities generally stabilized with greater numbers of reviews and that online ratings corresponded more closely to CMS survey ratings with greater numbers of reviews.^8,10^ To maximize facility inclusion while acknowledging these previous findings, we chose to limit facilities to those with at least 3 reviews, which was consistent with approaches used in previous work.^7,8^ For facilities with at least 3 reviews between 2015 and 2019, all reviews within that 5-year period were included in subsequent analyses. We conducted a sensitivity analysis using a 5-review cutoff for facility inclusion (eTable 2 in the Supplement). The final set of facilities were geocoded and linked to their respective counties. Facility inclusion and exclusion criteria are shown in the eFigure in the Supplement.

### Variables

Mean ratings were aggregated across facilities by county. To measure the association between facility ratings and mortality by county, we obtained 2018 county-level age-adjusted mortality data from the Centers for Disease Control and Prevention Wide-ranging Online Data for Epidemiological Research (WONDER) database.^1^ We obtained county-level variables associated with health outcomes from the University of Wisconsin School of Medicine and Public Health County Health Rankings (CHR) database.^19^ We used CHR composite measures for health behaviors, clinical care, social and economic factors, and physical environment. Descriptions of the variables used to calculate these composite measures can be found on the CHR website,^19^ and variables included data from January 1, 2014, to December 31, 2018.^3^ Scores were based on state quartiles (with quartile 1 indicating best health and quartile 4 indicating worst health) rather than state rankings to account for the unequal number of counties across states.

### Statistical Analysis

Mean age-adjusted mortality in included and excluded counties was compared using a 2-sided *t* test. The distribution of CHR state-level quartiles between included and excluded counties was compared using χ^2^ testing. A linear mixed-effects model was used to estimate the association between mortality and 2015 to 2019 mean annual facility rating by county. In our final model, we adjusted for county-level CHR composite scores across the 4 categories (health behaviors, clinical care, social and economic factors, and physical environment). The use of mixed-effects models allowed for the inclusion of random effects to adjust for nonindependence in hierarchical data. To adjust for possible state-level variation in the association between mean facility ratings and age-adjusted mortality by county, we included US states as a random effect. To assess whether the association was altered by the number of facilities in a county, we repeated our analysis for counties with 1 or more, 3 or more, and 5 or more facilities meeting inclusion criteria. We also repeated our analysis after increasing the minimum number of reviews needed for a facility to meet inclusion criteria from 3 reviews to 5 reviews. Hypothesis tests were 2-sided, and we considered *P* < .05 to be statistically significant.

To better understand how the language content of equally rated reviews might differ by county-level mortality, we conducted a natural language processing analysis comparing word frequencies across 4 review categories.^20,21,22,23^ We compared the frequency of words used across 5-star (maximum) and 1-star (minimum) reviews from a random subset of 10 000 facilities in the lowest mortality quartile and 10 000 facilities in the highest mortality quartile (among those counties meeting inclusion criteria). We removed all stop words, which are common words such as *the* and *if* that are unlikely to impart meaning, and we excluded words appearing in fewer than 5% of reviews in any of the 4 categories (ie, 1-star reviews in low-mortality counties, 1-star reviews in high-mortality counties, 5-star reviews in low-mortality counties, and 5-star reviews in high-mortality counties). Words were merged across verb tenses and plural forms. We compared the frequencies of words in the 4 categories using χ^2^ tests with an α of .001. We adjusted for multiple comparisons using the Bonferroni correction, consistent with other natural language processing analyses.^23^ All statistical analyses were performed using R software, version 3.6.1 (R Foundation for Statistical Computing).

## Results

Of 96 724 facilities meeting inclusion criteria, 95 120 (98.3%) were successfully geocoded to 1301 of 3142 US counties (41.4%) (eFigure in the Supplement). Counties had a median of 5 facilities (IQR, 2-27 facilities) that met inclusion criteria and a median of 27 reviews (IQR, 7-179 reviews). The mean (SD) county-level rating across the study period was 2.9 (0.7) stars. The 1301 counties that met inclusion criteria generally had lower age-adjusted mortality than the 1841 counties that were excluded (mean [SD], 781.3 [142.0] deaths per 100 000 people vs 852.6 [166.6] deaths per 100 000 people, respectively; *P* < .001) (Table 1). Compared with excluded counties, included counties were also more commonly in their states’ healthier quartiles for health behaviors (eg, 327 counties [17.8%] vs 448 counties [34.4%] in quartile 1, respectively; *P* < .001), clinical care (eg, 214 counties [11.6%] vs 561 counties [43.1%] in quartile 1; *P* < .001), and social and economic factors (eg, 319 counties [17.3%] vs 456 counties [35.0%] in quartile 1; *P* < .001) but in less healthy quartiles for physical environment (eg, 316 counties [17.2%] vs 458 counties [35.2%] in quartile 4; *P* < .001).

**Table 1.** All-Cause Mortality and County Health Ranking Variables in Included vs Excluded US Counties

Variable	Counties, No. (%)		*P* value
	Included (n = 1301)^a^	Excluded (n = 1841)^b^	
Age-adjusted deaths per 100 000 people, mean (SD)^c^	781.3 (142.0)	852.6 (166.6)	<.001
CHR variable by quartile^d^			
Health behaviors			
Quartile 1	448 (34.4)	327 (17.8)	<.001
Quartile 2	318 (24.4)	455 (24.7)	
Quartile 3	271 (20.8)	491 (26.7)	
Quartile 4	264 (20.3)	510 (27.7)	
Clinical care			
Quartile 1	561 (43.1)	214 (11.6)	<.001
Quartile 2	345 (26.5)	428 (23.2)	
Quartile 3	230 (17.7)	532 (28.9)	
Quartile 4	165 (12.7)	609 (33.1)	
Social and economic factors			
Quartile 1	456 (35.0)	319 (17.3)	<.001
Quartile 2	337 (25.9)	436 (23.7)	
Quartile 3	288 (22.1)	474 (25.7)	
Quartile 4	220 (16.9)	554 (30.1)	
Physical environment			
Quartile 1	222 (17.1)	553 (30.0)	<.001
Quartile 2	273 (21.0)	500 (27.2)	
Quartile 3	348 (26.7)	414 (22.5)	
Quartile 4	458 (35.2)	316 (17.2)	

Abbreviation: CHR, County Health Rankings database.

^a^ Included counties were those with ≥1 reviewed facility that met inclusion criteria.

^b^ Excluded counties were those with 0 reviewed facilities that met inclusion criteria.

^c^ Available for 3058 of 3142 US counties (97.3%) and all 1301 counties with at least 1 reviewed facility that met inclusion criteria. Mean age-adjusted mortality per 100 000 people was compared using a 2-sided *t* test.

^d^ Quartile 1 indicates best health, and quartile 4 indicates worst health. Quartiles were defined by state. Distributions of state quartiles were compared using an χ^2^ test.

**Figure 1.**
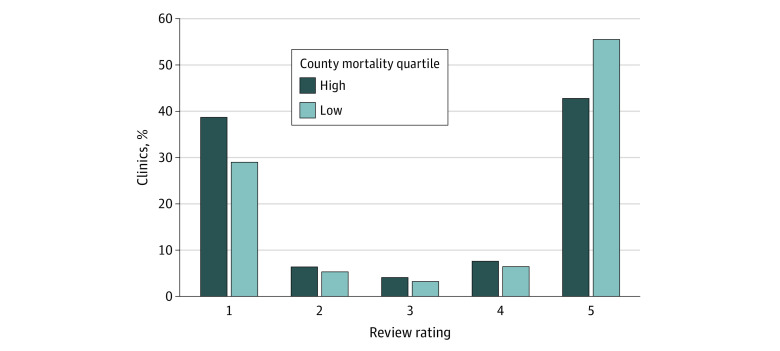
Yelp Rating Distribution for Counties in the Lowest and Highest Mortality Quartiles Among counties with at least 1 reviewed essential health care facility.

The distribution of ratings across all facilities was bimodal (ie, 1-star vs 5-star); of 1 242 317 total reviews, 413 921 (33.3%) were 1-star reviews, and 656 679 (52.9%) were 5-star reviews. Compared with facilities in counties with the highest mortality, those in counties with the lowest mortality received more 5-star ratings (42.9% vs 55.6%, respectively; *P* < .001) and fewer 1-star ratings (38.8% vs 29.1%; *P* < .001) (Figure 1).

In the final adjusted model, a 1-point increase in mean rating among all counties with 1 or more reviewed facilities (n = 1301) was associated with a mean (SE) age-adjusted decrease of 18.05 (3.68) deaths per 100 000 people (95% CI, 10.83-25.28 deaths per 100 000 people; *P* < .001) (Table 2). When restricted to counties with 3 or more reviewed facilities (n = 848), the mean (SE) age-adjusted decrease was 52.72 (6.74) deaths per 100 000 people (95% CI, 39.48-65.96 deaths per 100 000 people; *P* < .001). When restricted to counties with 5 or more reviewed facilities (n = 686), the mean (SE) age-adjusted decrease was 60.94 (8.78) deaths per 100 000 people (95% CI, 43.70-78.17 deaths per 100 000 people; *P* < .001). This age-adjusted decrease in mortality was also greater when the analysis was limited to facilities with 5 or more reviews, as compared with the baseline of 3 or more reviews (eTable 2 in the Supplement). In the final adjusted model including counties with 1 or more reviewed facilities having 5 or more reviews (n = 934), a 1-point increase in mean rating was associated with a mean (SE) decrease of 25.16 (4.67) deaths per 100 000 people (95% CI, 15.99-34.33 deaths per 100 000 people; *P* < .001).

**Table 2.** Linear Regression Analysis of 2018 All-Cause Mortality

Variable	Null model (n = 1301)^a^		Complete models^a^					
			Counties with ≥1 reviewed facility (n = 1301)		Counties with ≥3 reviewed facilities (n = 848)		Counties with ≥5 reviewed facilities (n = 686)	
	Age-adjusted deaths per 100 000 people (SE)	95% CI	Age-adjusted deaths per 100 000 people (SE)	95% CI	Age-adjusted deaths per 100 000 people (SE)	95% CI	Age-adjusted deaths per 100 000 people (SE)	95% CI
Mean platform rating, 2015-2019	−41.30 (4.60)	−50.32 to −32.27	−18.05 (3.68)	−25.28 to −10.83	−52.72 (6.74)	−65.96 to −39.48	−60.94 (8.78)	−78.17 to −43.70
CHR variable by quartile^b^								
Health behaviors								
Quartile 1	NA	NA	1 [Reference]	NA	1 [Reference]	NA	1 [Reference]	NA
Quartile 2	NA	NA	63.58 (7.25)	49.37 to 77.80	59.72 (8.11)	43.80 to 75.63	60.08 (8.55)	43.28 to 76.88
Quartile 3	NA	NA	99.70 (8.21)	83.60 to 115.81	99.75 (9.61)	80.90 to 118.61	103.49 (10.20)	83.46 to 123.51
Quartile 4	NA	NA	142.09 (9.41)	123.63 to 160.54	153.60 (11.34)	131.33 to 175.86	165.19 (12.31)	141.00 to 189.37
Clinical care								
Quartile 1	NA	NA	1 [Reference]	NA	1 [Reference]	NA	1 [Reference]	NA
Quartile 2	NA	NA	19.24 (6.60)	6.29 to 32.19	20.52 (7.48)	5.83 to 35.20	16.98 (7.96)	1.36 to 32.60
Quartile 3	NA	NA	14.19 (7.77)	−1.06 to 29.43	8.30 (9.44)	−10.23 to 26.84	−2.70 (10.28)	−22.89 to 17.48
Quartile 4	NA	NA	16.21 (9.23)	−1.91 to 34.33	17.04 (11.55)	−5.63 to 39.72	−13.74 (12.72)	−38.70 to 11.23
Social and economic factors								
Quartile 1	NA	NA	1 [Reference]	NA	1 [Reference]	NA	1 [Reference]	NA
Quartile 2	NA	NA	26.44 (7.24)	12.24 to 40.64	13.36 (8.17)	−2.67 to 29.40	11.66 (8.53)	−5.09 to 28.41
Quartile 3	NA	NA	34.59 (8.31)	18.29 to 50.89	21.09 (9.90)	1.65 to 40.52	18.62 (10.56)	−2.11 to 39.35
Quartile 4	NA	NA	63.53 (10.09)	43.74 to 83.32	32.82 (12.05)	9.17 to 56.46	21.22 (12.92)	−4.15 to 46.58
Physical environment								
Quartile 1	NA	NA	1 [Reference]	NA	1 [Reference]	NA	1 [Reference]	NA
Quartile 2	NA	NA	10.17 (8.27)	−6.06 to 26.40	22.52 (10.07)	2.75 to 42.30	12.33 (10.74)	−8.77 to 33.43
Quartile 3	NA	NA	−0.11 (7.87)	−15.55 to 15.33	5.37 (9.45)	−13.19 to 23.92	3.69 (10.02)	−15.99 to 23.37
Quartile 4	NA	NA	8.38 (7.62)	−6.56 to 23.32	12.05 (9.45)	−6.50 to 30.59	7.71 (10.07)	−12.06 to 27.48

Abbreviations: CHR, County Health Rankings database; NA, not applicable.

^a^ All models include state-level random effects.

^b^ Quartile 1 indicates best health, and quartile 4 indicates worst health. Quartiles were defined by state.

**Figure 2.**
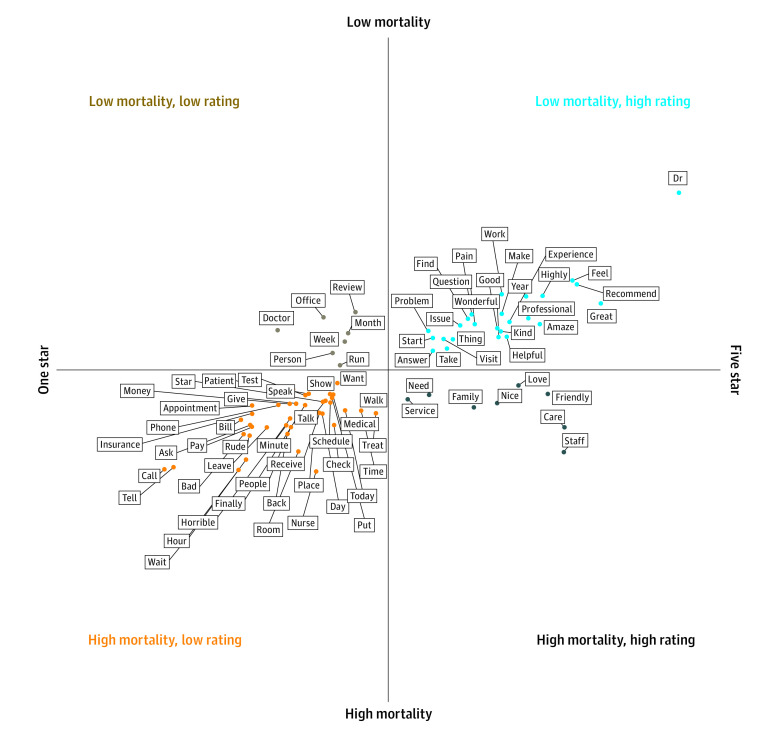
Words With Significantly Different Frequency of Use Among 5-Star and 1-Star Reviews of Facilities in Counties With the Lowest and Highest Mortality Quartiles

Among 1-star reviews, words related to time (eg, *minute[s]*, *hour[s]*, *finally*, *wait[ing]*, and *schedule[s]*) were more common in counties with high mortality, as were words related to payment (eg, *money*, *insurance*, and *pay*) and interpersonal interactions (eg, *rude* and *tell* or *told*). Among 5-star reviews, several words, including *friendly*, *nice*, and *staff*, were more common in counties with high mortality, whereas other words, including *pain*, *question*, *helpful*, and *Dr*, were more common in counties with low mortality (Figure 2).

## Discussion

This cross-sectional study had 2 main findings. First, among counties with rated essential health care facilities, lower facility ratings were associated with higher mortality. Second, 1-star (lowest rating) and 5-star (highest rating) reviews of facilities in counties with high vs low mortality used substantially different language when describing experiences of care. Counties with at least 1 rated health facility that met inclusion criteria were generally healthier than counties with no rated facilities, as observed in lower mean age-adjusted mortality and overrepresentation of counties in their states’ healthiest quartile of health behaviors, clinical care, and social and economic factors. This finding may reflect more frequent use of the online platform’s review site in urban areas. In the US, mortality rates are generally lower in urban vs rural counties, and this mortality gap has been increasing.^24^ Health care facilities and practitioners, such as primary care physicians, are also in lower supply per capita in rural counties and are less densely distributed.^25^

The negative association between mean facility rating and mortality suggests either that those living in counties with higher mortality are more likely to rate a comparable health care facility unfavorably or that facilities in counties with higher mortality are more likely to provide worse care. The findings of previous studies favor the latter hypothesis, reporting that patient satisfaction is largely associated with patients’ experiences of communication with hospital staff as well as patient- and hospital-level health outcomes.^4,5,6^ In contrast, data regarding the association between disease severity and patient satisfaction have been mixed.^26^ In the present study, the association between county-level mortality and local reviews of essential health care facilities was greater when the analysis was restricted to counties with multiple rated facilities, which supports our hypothesis that a true association exists between patients’ experiences of care and health outcomes; if low ratings of essential health care facilities were associated with worse local health outcomes, we would expect counties with multiple low-rated facilities to have worse health outcomes than those with 1 low-rated facility (if all other variables were equal).

Although facilities in both high- and low-mortality counties had large numbers of 5-star and 1-star reviews, the language used in equivalently rated reviews varied by county-level mortality. These differences in review language across facilities in high-mortality vs low-mortality counties are hypothesis-generating, suggesting areas of focus for improving health care satisfaction in high-mortality counties. For instance, more frequent use of the words *tell* or *told* and *rude* among 1-star reviews in high-mortality counties may suggest less patient-centered communication in such settings.^22^ Patient-centered communication has been associated with improvements in patient satisfaction, medication adherence, and health outcomes.^27^ One study of adults enrolled in a public health program found that perceived discrimination was significantly associated with preventive care that was delayed or not received.^28^ Words related to cost and payment were also more frequent in reviews of facilities in high-mortality counties. Cost has been consistently identified as a barrier to health care use, and high health care costs have disproportionate consequences for those living in poverty.^28,29^ Counties with lower median household incomes and more residents with income lower than the poverty line have higher mortality rates compared with other US counties.^2^ In addition, words emphasizing time (eg, *hour[s]* and *wait*) may reflect both more difficulty in accessing timely care in high-mortality counties and an unequal distribution of time as a commodity, with those who have low-wage jobs being less able to afford time off from work, for instance. In emergency departments, longer waiting times and lengths of stay may be associated with a greater risk of death and hospital admission.^30,31^

In low-mortality counties, the words most frequently used in 1-star and 5-star reviews suggest more personal relationships with health care professionals and different experiences with timeliness of care. The word *Dr*, presumably followed by a surname, was most specific to 5-star reviews in low-mortality counties, suggesting a potentially greater familiarity and comfort between patients and physicians in such settings. Among 1-star reviews of facilities in low-mortality counties, time-related words referred to longer periods (eg, *week[s]* and *month[s]*) compared with time-related words specific to 1-star reviews of facilities in high-mortality counties. Longer periods may reflect longer waiting times to obtain an appointment rather than longer waiting times after arrival at a health care facility. Data from US Department of Veterans Affairs medical centers supports an association between long waiting times for primary care appointments and both hospitalization and mortality.^31,32^ Consistent references to time across 1-star reviews in both high- and low-mortality counties suggest that efforts to decrease waiting times to obtain both urgent and preventive health care may have benefits for patient care experiences and health outcomes.

### Limitations

This study has several limitations. First, we do not know the specific demographic characteristics of the reviewers of health care facilities on the online platform, nor do we know how those data compare with overall county demographic characteristics. This platform is more commonly used by those with at least some college education and higher income, both of which have been significantly associated with lower mortality.^33,34^ Our study may therefore overrepresent groups with lower mortality across both high- and low-mortality counties. The platform is also used equally among those aged 18 to 34 years (31%), 35 to 54 years (34%), and 55 years and older (33%).^33^ Given that health care use typically increases with increasing age, data from the platform may underrepresent older adults. It is, however, possible that some younger adults on the platform are posting reviews about their experiences with facilities providing care to their older relatives. We were also unable to account for the possibility that multiple facility reviews were written by the same user or family, although the platform does filter reviews for authenticity, both manually and algorithmically, which decreases the risk of repeat posts.^35^

Second, we were limited to relying on platform developer tags to identify facility types because we had no official list of facilities providing essential health care. Data on facilities from many categories of essential health care, including preventive and prescription drug services, are not available through CMS databases. As described in Methods, we also chose to analyze the data in aggregate across all platform categories matched to essential health benefits. Future analyses may benefit from exploring the association between online reviews for specific subgroups of essential health care facilities and local health outcomes in addition to specific county subgroups (eg, rural vs urban counties and low-income vs high-income counties).

Third, the direction of the association between mortality and patient-reported experiences of care is not clear, and unmeasured variables may alter this association. One previous cross-sectional study examining hospital- and county-level variables found that 27% of the variation in CMS hospital ratings could be explained by county characteristics.^36^ For instance, hospitals with higher ratings were more likely to be located in counties with fewer Black and Latinx residents and more primary care physicians per capita.^36^ Our findings warrant further exploration of patient-, provider-, and community-level factors associated with unfavorable health outcomes and low care satisfaction as well as independent attention to health care satisfaction as an outcome in itself.

## Conclusions

To our knowledge, this cross-sectional study is the first to describe the association between county-level crowdsourced ratings of health care and health outcomes. A negative association was found between local essential health care facility ratings and county-level mortality. Furthermore, even among reviews with equivalent ratings, natural language processing analyses revealed important differences in review content between counties with high vs low mortality. Equivalent online ratings did not necessarily reflect equivalent experiences of care across counties with different mortality levels, as evidenced by variations in the frequency of use of key words in reviews. Future analyses may benefit from exploring the factors associated with decreased health care satisfaction among patients in high-mortality counties. Improving patient satisfaction has the potential to improve community trust in health care and community-level health outcomes.

## Notes

Supplement. **eTable 1.** Mapping Yelp Health Categories to ACA 10 Essential Health Benefits **eTable 2.** Linear Regression Model Results for 2018 Age-Adjusted All-Cause Mortality per 100 000 People in Counties With at Least 1 Yelp Facility Meeting Inclusion Criteria and at Least 5 Reviews **eFigure.** Facilities Eliminated by Exclusion Criteria and Number and Percentage of Successfully Geocoded Facilities Meeting Inclusion Criteria
