# Supplementary material for: Association Between Crowdsourced Health Care Facility Ratings and Mortality in US Counties
Source: JAMA Netw Open. 2021 Oct 19;4(10):e2127799. doi: 10.1001/jamanetworkopen.2021.27799 (PMC8527362; doi:10.1001/jamanetworkopen.2021.27799)
Supplement: Supplement. — eTable 1. Mapping Yelp Health Categories to ACA 10 Essential Health Benefits eTable 2. Linear Regression Model Results for 2018 Age-Adjusted All-Cause Mortality per 100 000 People in Counties With at Least 1 Yelp Facility Meeting Inclusion Criteria and at Least 5 Reviews eFigure. Facilities Eliminated by Exclusion Criteria and Number and Percentage of Successfully Geocoded Facilities Meeting Inclusion Criteria [file jamanetwopen-e2127799-s001.pdf]

Supplementary Online Content

Stokes DC, Pelullo AP, Mitra N, et al. Association between crowdsourced health care facility ratings and mortality in US counties. *JAMA Netw Open*. 2021;4(10):e2127799. doi:10.1001/jamanetworkopen.2021.27799

eTable 1. Mapping Yelp Health Categories to ACA 10 Essential Health Benefits

eTable 2. Linear Regression Model Results for 2018 Age-Adjusted All-Cause Mortality per 100,000 People in Counties With at Least 1 Yelp Facility Meeting Inclusion Criteria and at Least 5 Reviews

eFigure. Facilities Eliminated by Exclusion Criteria and Number and Percentage of Successfully Geocoded Facilities Meeting Inclusion Criteria

This supplementary material has been provided by the authors to give readers additional information about their work.

© 2021 Stokes DC et al. *JAMA Network Open*.

eTable 1. Mapping Yelp Health Categories to ACA 10 Essential Health Benefits

ACA 10 Essential Health Benefit(s)	Yelp categories	Percent of facilities meeting inclusion criteria (n=95,120) with at least one Yelp category corresponding to ACA Essential Health Benefit
1) Ambulatory patient services (outpatient care you get without being admitted to a hospital)<br>2) Hospitalization (like surgery and overnight stays)<br>3) Preventive and wellness services and chronic disease management	Allergists, Anesthesiologists, Cardiologists, Dermatologists, Dialysis Clinics, Dietitians, Ear Nose & Throat, Endocrinologists, Family Practice, Gastroenterologist, Gerontologists, Hepatologists, Hospice, Hospitalists, Hospitals, Immunodermatologists, Infectious Disease Specialists, Internal Medicine, Medical Centers, Nephrologists, Neurologist, Neuropathologists, Neurotologists, Nurse Practitioner, Nutritionists, Oncologist, Ophthalmologists, Orthopedists, Osteopathic Physicians, Osteopaths*, Otologists, Pain Management, Pathologists, Doctors, Plastic Surgeons, Podiatrists, Preventive Medicine, Proctologists, Pulmonologist, Retina Specialists, Rheumatologists, Sleep Specialists, Spine Surgeons, Sports Medicine, Surgeons, Undersea/Hyperbaric Medicine, Urgent Care, Urologists, Vascular Medicine, Walk-in Clinics	65. 4%
4) Emergency services	Emergency Medicine, Emergency Rooms, Toxicologists	0.7%
5) Pregnancy, maternity, and newborn care	Lactation Services, Midwives, Obstetricians & Gynecologists, Postpartum Care*, Prenatal/Perinatal Care	6.3%
6) Mental health and substance use disorder services, including behavioral health treatment (this includes counseling and psychotherapy)	Addiction Medicine, Counseling & Mental Health, Halfway Houses, Psychiatrists, Psychologists, Psychotherapists*	5.8%
7) Prescription drugs	Pharmacy	6.8%
8) Rehabilitative and habilitative services and devices (services and devices to help people with injuries, disabilities, or chronic conditions gain or recover mental and	Habilitative Services, Home Health Care, Occupational Therapy, Physical Therapy, Rehabilitation Center, Skilled Nursing	13.2%

© 2021 Stokes DC et al. *JAMA Network Open*.

physical skills)		
9) Laboratory services	Diagnostic Imaging, Diagnostic Services, Laboratory Testing, Phlebologists, Radiologists, Ultrasound Imaging Centers	5.9%
10) Pediatric services, including oral and vision care (but adult dental and vision coverage aren't essential health benefits)	Pediatricians, Speech Therapists	5.4%
Yelp categories deemed unlikely to be covered by ACA 10 Essential Health Benefits	Acupuncture, Aestheticians, Alternative Medicine, Animal Assisted Therapy, Assisted Living Facilities, Audiologist, Ayurveda, Behavior Analysts, Blood & Plasma Donation Centers, Body Contouring, Bulk Billing*, Cannabis Clinics, Cannabis Collective, Medical Cannabis Referrals, Cannabis Tours, Chiropractors, Colonics, Concierge Medicine, Cosmetic Dentists, Cosmetic Surgeons, Crisis Pregnancy Centers, Cryotherapy, Storefront Clinics (Dental Hygienists)*, Dental Hygienists, Mobile Clinics (Dental Hygienists)*, Dentists, Doulas, Endodontists, Faith-based Crisis Pregnancy Centers, Fertility, Float Spa, General Dentistry, Geneticists, Halotherapy, Health & Medical, Health Coach, Health Insurance Offices, Hearing Aids, Hearing Aid Providers, Herbal Shops, Homeopathic*, Hydrotherapy, Hypnosis/Hypnotherapy, IV Hydration, Laser Eye Surgery/Lasik, Lice Services, Massage Therapy, Medical Foot Care*, Medical Spas, Medical Transportation, Memory Care, Naturopathic/Holistic, Optometrists, Oral Surgeons, Organ & Tissue Donor Services, Orthodontists, Orthotics, Oxygen Bars, Pediatric Dentists, Periodontists, Personal Care Services, Placenta Encapsulations, Prosthetics, Prosthodontists, Psychoanalysts, Psychotechnical Tests, Reflexology, Reiki, Reproductive Health Services, Retirement Homes, Saunas, Sex Therapists, Sophrologists*, Sperm Clinic, Sports Psychologists, Tattoo Removal, Traditional Chinese Medicine, Tropical Medicine*, Tui Na, Weight Loss Centers	Excluded

*Category exists in Yelp but was not attributed to any facilities in the sample

© 2021 Stokes DC et al. *JAMA Network Open*.

eTable 2. Linear Regression Model Results for 2018 Age-Adjusted All-Cause Mortality per 100,000 People in Counties With at Least 1 Yelp Facility Meeting Inclusion Criteria and at Least 5 Reviews (n = 934)

Variable	Null model		Complete model with County Health Rankings control variables	
**Yelp Variables**	Model coefficient estimate (SE)[a]	95% CI	Model coefficient estimate (SE)[a]	95% CI
2015-2019 mean review rating	-56.88 (5.86)	-68.38 to -56.88	-25.16 (4.67)	-34.33 to -15.99
**County Health Rankings Variables[b]**				
"Health Behaviors"				
1st Quartile [Reference]				
2nd Quartile			62.62 (7.95)	47.02 to 78.22
3rd Quartile			98.75 (9.19)	80.71 to 116.8
4th Quartile			155.04 (10.84)	133.75 to 176.32
"Clinical Care"				
1st Quartile [Reference]				
2nd Quartile			23.19 (7.20)	9.07 to 37.32
3rd Quartile			3.96 (8.94)	-13.59 to 21.51
4th Quartile			16.69 (10.71)	-4.33 to 37.72
"Social and Economic Factors"				
1st Quartile [Reference]				
2nd Quartile			23.82 (7.96)	8.19 to 39.43
3rd Quartile			26.01 (9.38)	7.6 to 44.41
4th Quartile			42.28 (11.49)	19.72 to 64.84

© 2021 Stokes DC et al. *JAMA Network Open*.

"Physical Environment"				
1st Quartile [Reference]				
2nd Quartile			29.71 (9.48)	11.1 to 48.31
3rd Quartile			11.80 (8.88)	-5.62 to 29.22
4th Quartile			22.23 (8.75)	5.04 to 39.41

[a]All models include state-level random effects

[b]Lower quartile indicates "healthier" county

© 2021 Stokes DC et al. *JAMA Network Open*.

eFigure. Facilities Eliminated by Exclusion Criteria and Number and Percentage of Successfully Geocoded Facilities Meeting Inclusion Criteria

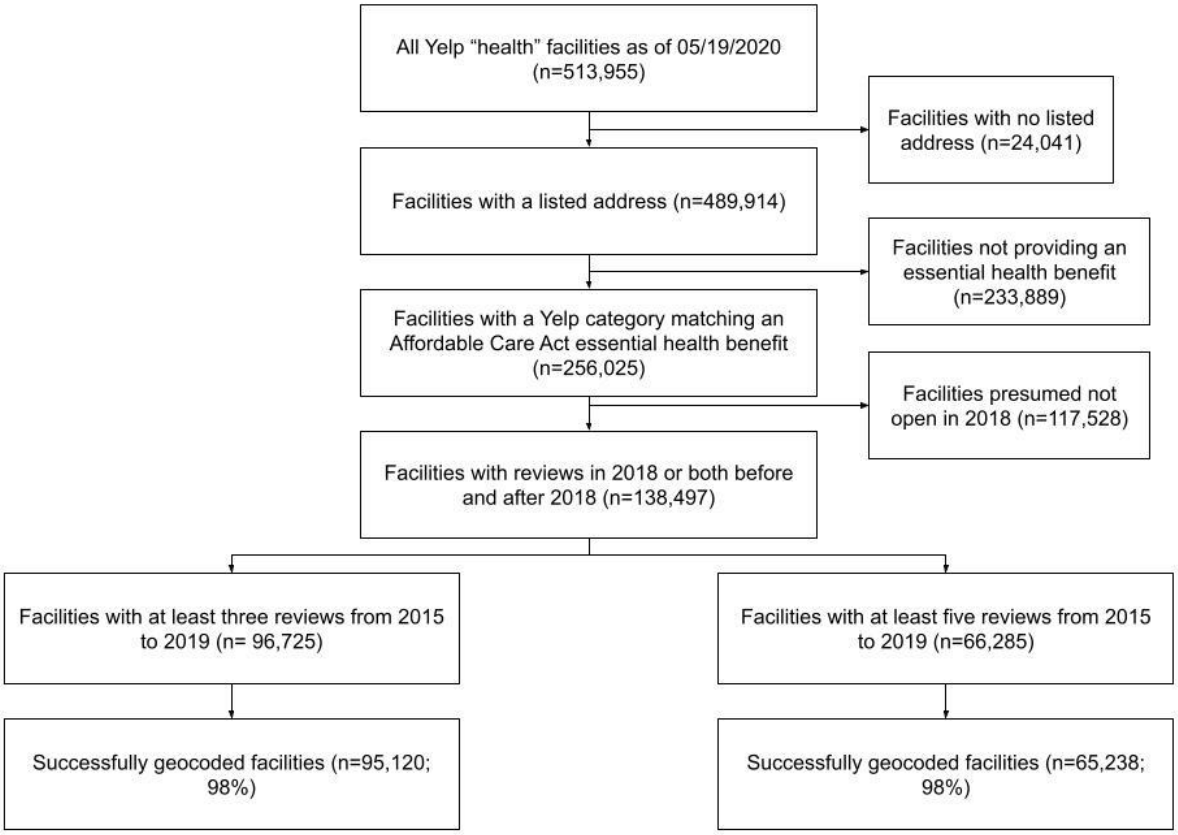

© 2021 Stokes DC et al. *JAMA Network Open*.
